# Enhanced Molecular Surveillance of Chikungunya Virus

**DOI:** 10.1128/mSphere.00295-19

**Published:** 2019-07-03

**Authors:** Carlo Fischer, Xavier de Lamballerie, Jan Felix Drexler

**Affiliations:** a Berlin Institute of Health, Institute of Virology, Charité-Universitätsmedizin Berlin (corporate member of Freie Universität Berlin, Humboldt-Universität zu Berlin), Berlin, Germany; b Unité des Virus Émergents (Aix-Marseille University, IRD 190, Inserm 1207, IHU Méditerranée Infection), Marseille, France; c German Centre for Infection Research; d Martsinovsky Institute of Medical Parasitology, Tropical and Vector-Borne Diseases, Sechenov University, Moscow, Russia; University of Texas Southwestern Medical Center

**Keywords:** adaptive mutations, alphavirus, molecular epidemiology, vector-borne diseases

## Abstract

Outbreaks of the emerging arbovirus chikungunya virus (CHIKV) affect millions of individuals in Asia, Africa, and Latin America. Vector competence can be changed dramatically by single amino acid exchanges located predominantly within the CHIKV E1 and E2 envelope proteins, which are associated with enhanced transmissibility by anthropophilic *Aedes* mosquitoes. Commonly used reference assays for molecular surveillance cover only a few adaptive mutations within the envelope domains and have not been validated for all CHIKV genotypes. The recognized landscape of CHIKV adaptive mutations is thus likely incomplete. We designed two nested reverse transcription-PCR (RT-PCR) assays that cover hot spots of viral adaptation to vectors within the E1 and E2 genomic domains. Primers were designed in conserved genomic regions to allow broad usability across CHIKV genotypes. The sensitivity of both assays was at least equivalent to E1- and E2-based reference assays and robust among CHIKV genotypes at 51.4 IU/reaction (E1, 95% confidence interval [CI], 39.8 to 78.9) and 4.0 IU/reaction (E2, 95% CI, 2.0 to 7.4). Upon analysis of the complete known CHIKV genomic diversity, up to 11 nucleotide mismatches with CHIKV variants occurred under oligonucleotide binding sites of reference assays, potentially limiting assay sensitivity, whereas no critical mismatches occurred in the new assays. Specificity testing with nine alphaviruses representing all serocomplexes showed amplification of Mayaro virus and O'nyong-nyong virus by the E1-based assay, but not by the E2-based assay. The high sensitivity and specificity of the new E2-based assay may allow its diagnostic usage in resource-limited settings. The combined new assays allow improved molecular epidemiological surveillance of CHIKV globally.

**IMPORTANCE** The life cycle of arboviruses relies on efficient infection of and transmission by arthropod vectors. Adaptation to new vectors can thus dramatically increase the geographic range of an arbovirus. Several adaptive mutations enhance chikungunya virus (CHIKV) transmissibility by different mosquito species. The appearance of those adaptive mutations has led to large-scale CHIKV outbreaks in Asia, Africa, and Europe. Molecular surveillance of circulating CHIKV strains for adaptive mutations contributes to precise risk assessments and efficient vector control and provides new insight into the evolution of vector adaptation. Existing assays for molecular CHIKV surveillance are limited by poor coverage of known adaptive mutations, low sensitivity, and cost-intensive deep sequencing approaches, preventing universal application. We developed two highly sensitive nested RT-PCR assays that cover hot spots of vector adaptation in CHIKV envelope domains. The new assays allow unprecedented molecular surveillance across all CHIKV genotypes and diagnostic use in resource-limited settings globally.

## Abstract (precis)

The life cycle of arboviruses relies on efficient infection of and transmission by arthropod vectors. Adaptation to new vectors can thus dramatically increase the geographic range of an arbovirus. Several adaptive mutations enhance chikungunya virus (CHIKV) transmissibility by different mosquito species. The appearance of those adaptive mutations has led to large-scale CHIKV outbreaks in Asia, Africa, and Europe. Molecular surveillance of circulating CHIKV strains for adaptive mutations contributes to precise risk assessments and efficient vector control and provides new insight into the evolution of vector adaptation. Existing assays for molecular CHIKV surveillance are limited by poor coverage of known adaptive mutations, low sensitivity, and cost-intensive deep sequencing approaches, preventing universal application. We developed two highly sensitive nested RT-PCR assays that cover hot spots of vector adaptation in CHIKV envelope domains. The new assays allow unprecedented molecular surveillance across all CHIKV genotypes and diagnostic use in resource-limited settings globally.

## OBSERVATION

Chikungunya virus (CHIKV) is an emerging arthropod-borne virus (arbovirus) with major impact on public health (1). Infection with this rapidly evolving RNA virus (2) commonly leads to severe polyarthralgia, frequently persisting for prolonged periods of time (1, 3). CHIKV has been classified into the Asian, the East/Central/South African (ECSA), and West African genotypes. Since 2005, CHIKV has spread extensively and is now endemic in large parts of Africa, Asia, Oceania, and South and North America (1, 4, 5). Transmissibility of CHIKV by anthropophilic Aedes aegypti or Aedes albopictus mosquitos can be enhanced dramatically by single amino acid exchanges (6, 7), increasing attack rates during outbreaks and expanding outbreaks to temperate climates upon adaptation to widespread Aedes albopictus mosquitoes (1). At least 14 adaptive mutations are known, most of which are located in the genomic domains encoding the viral envelope proteins E2 and E1 (7–13). Primary adaptations increase the transmission by mosquito vectors, secondary adaptations provide additional fitness gains in the presence of a primary adaptation, and finally some amino acid exchanges exert epistatic effects favoring or preventing the occurrence of primary or secondary adaptations (5, 7). The impact of adaptive mutations is highlighted by the explosive outbreaks caused by the CHIKV ECSA Indian Ocean lineage (IOL) affecting up to 10 million individuals during 2005 to 2007 (7, 14). Molecular epidemiological studies revealed that the outbreak magnitude was associated with efficient transmission of the IOL by Aedes albopictus, which was mediated by two adaptive primary mutations, E2-L210Q and E1-A226V (10). Surveillance of adaptive mutations in circulating CHIKV lineages is therefore crucial for risk assessments and vector control strategies (15). However, existing reverse transcription-PCR (RT-PCR) assays for molecular CHIKV surveillance cover a very limited number of the known adaptive mutations. Full-genome-based approaches are an alternative, but are cost and labor intensive, not universally available, and technically challenging in specimens with low viral load: e.g., taken late during the viremic phase (16, 17). To improve molecular CHIKV surveillance, we designed new RT-PCR assays amenable for usage across all CHIKV genotypes and in resource-limited settings.

## 

### Observations.

**FIG 1:**
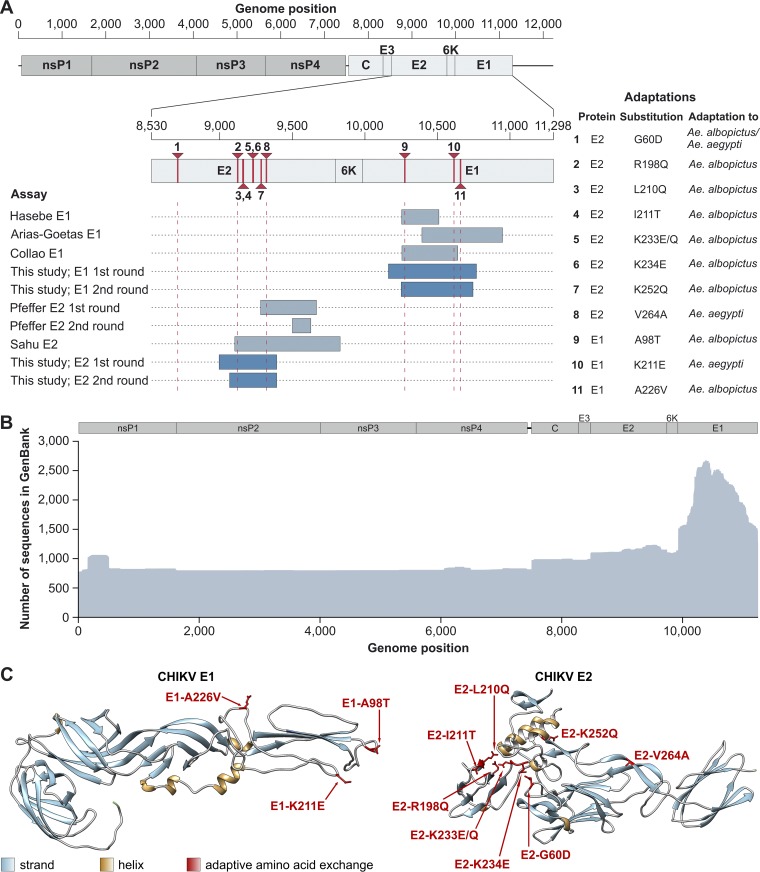
Adaptive amino acid exchanges in CHIKV and assay design. (A) Genomic localization of adaptive mutations and commonly used PCR assays. Genome positions are shown for a CHIKV reference genome (GenBank accession no. MG967666). Positions of adaptive mutations are indicated in red and by vertical dotted red lines at their extremities. (B) Sequence coverage and identity of the coding region of the CHIKV genome based on all available GenBank entries (18 March 2019). Coverage and identity were calculated using Geneious 9.1.8 and were visualized using R version 3.5.2. (C) Tridimensional localization of known adaptive amino acid exchanges in the CHIKV E1 and E2 proteins. The amino acid exchanges E1-T98A (7), E1-K211E (13), E1-A226V (7), E2-G60D (9), E2-R198Q (7), E2-L210Q (7), E2-I211T (9), E2-K233E/Q (7), E2-K234E (7), E2-K252Q (7), and E2-V264A (12) are depicted.

Of the known adaptive mutations, three are located in the E1 genomic domain and eight in the E2 genomic domain (Fig. 1A). Nevertheless, the sequence coverage of the E1 domain by GenBank entries is 2-fold higher than for the E2 domain (Fig. 1B). The relative abundance of E1 sequences is likely due to the biological relevance of the E1 substitution A226V, but also due to the unavailability of suitable tools since the majority of assays commonly used for CHIKV surveillance target small parts of the E1 domain only (Fig. 1A). Broadly used E2-based assays are hardly available and frequently do not cover all relevant adaptive mutations (Fig. 1A).

To illustrate the three-dimensional localization of the known adaptive substitutions, we analyzed the crystal structures of the E1 and E2 proteins. The adaptive substitutions are not spread randomly across the CHIKV envelope proteins. Within E2, six substitutions are in close three-dimensional proximity (Fig. 1C), including a small region ranging from amino acid residues 210 to 252, which interacts with the fusion loop of E1, mediating virus entry by fusion of viral and cellular membranes (7). Within the E1 protein, three substitutions are spread over 128 amino acid residues but are localized in close three-dimensional proximity (Fig. 1C). The three-dimensional proximity of adaptive substitutions may suggest that future amino acid exchanges affecting vector competence could also be localized within these domains, which should thus be optimal candidates for the development of assays amenable for sustainable use.

We designed two new nested RT-PCR assays targeting these CHIKV E2 and E1 domains. The assay design was done based on 1,763 GenBank entries covering at least partially the E2 or E1 domain of CHIKV. Among other reasons, RNA secondary structures such as those involved in viral replication and essential protein domains such as active sites of viral enzymes or viral domains interacting with cellular components prevent mutations from being equally distributed across virus genomes. It is thus more likely that future mutations will occur at positions that already show genetic variation between known CHIKV sequences (18, 19). Considering the complete genetic CHIKV diversity for primer design thus increases the robustness of the assays for all known strains but also for CHIKV strains emerging in the future. While the original intention was to cover the complete E2-E1 region, insufficient sensitivity and insufficient robustness across all CHIKV genotypes forced us to target smaller subgenomic domains and test different primer combinations to achieve adequate assay sensitivity.

**FIG 2:**
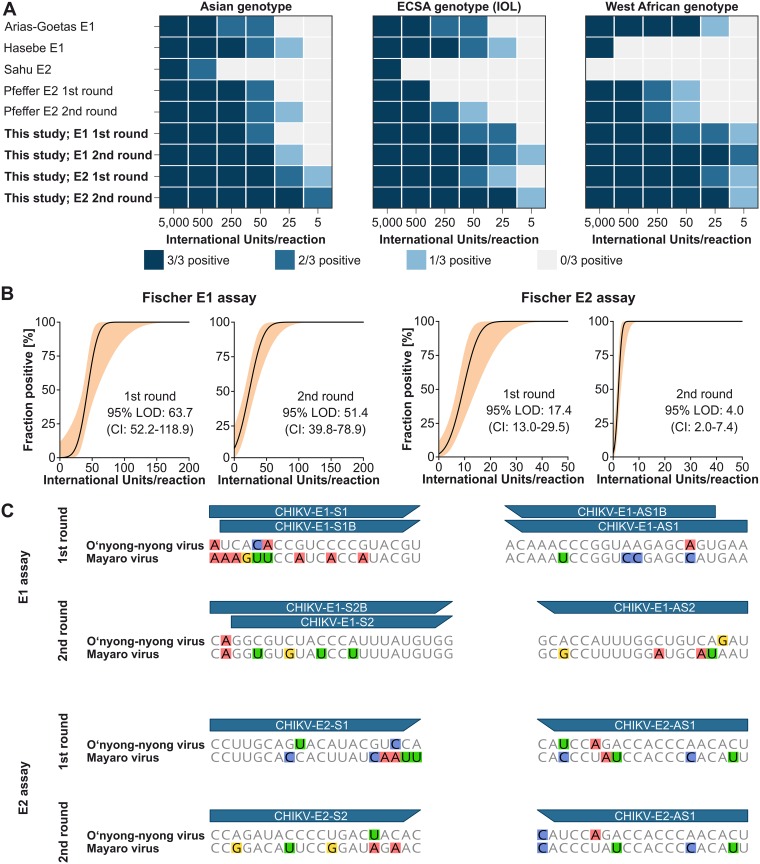
Assay validation. (A) Determination of robustness across CHIKV genotypes. New assays (boldface) and reference assays were tested with CHIKV RNA of the Asian, ECSA, and West African genotypes. (B) Determination of lower limits of detection (LOD) for the new PCR assays. Quantified RNA of the Asian CHIKV genotype was diluted stepwise and was tested in eight replicates with first-round assays. Probit analyses were done using R 3.5.2. (C) Conservation of primer target regions among representative Mayaro (NC003417) and O'nyong-nyong (NC001512) viruses. Mismatches with the new CHIKV assays are highlighted.

The oligonucleotide primers and protocol finally used cover all but one of the known adaptive mutations in the envelope domains and are shown in Table 1. RNA of all three CHIKV genotypes was tested with the new assays and commonly used E1-based (20–23) or E2-based (11, 24) reference assays. The new assays amplified all three CHIKV genotypes robustly, whereas reference assays showed limited sensitivity in at least one genotype (Fig. 2A). Variable sensitivity of reference assays was consistent with at least one primer used in those assays that contains possible mismatches with one or more CHIKV genotypes at the critical 3′ end of primers affecting assay sensitivity the most (18) (see Fig. S1 in the supplemental material). We added nested PCR rounds to ensure high sensitivity even for low-titer specimens (Fig. 2A). The exact lower limits of detection (LOD) were assessed using RNA dilutions of the Asian CHIKV genotype quantified using the recently developed WHO international standard. Nested PCR improved the LOD about 4-fold for the E2-based assay, whereas the LOD of the E1-based assay was only mildly improved. In contrast to E2, E1-based typing may thus benefit only marginally from a second round of PCR. The 95% LOD for the E1-based assay were 63.7 IU/reaction (95% confidence interval [CI], 52.2 to 118.9) in the first round and 51.5 IU/reaction (95% CI, 39.8 to 78.9) in the second round. The 95% LOD for the E2-based assay were 17.4 IU/reaction (95% CI, 13.0 to 29.5) in the first round and 4.0 IU/reaction (95% CI, 2.0 to 7.4) in the second round (Fig. 2B). Nested PCR increases the risk of amplicon contamination. Standard precautions should therefore be applied: e.g., handling of reagents and of first and second round PCR products and amplicon visualization in different areas.

**TABLE 1:** Assays and primers used in this study^*a*^

Assay	Primer	Genome position	5′→3′ sequence^*b*^	Concn (nM)
E1 assay				
1st round (649 bp)	CHIKV-E1-S1	10141–10160	GTYATCCCSTCYCCGTACGT	480
	CHIKV-E1-S1B	10142–10160	TYATCCCGTCTCCGTACGT	200
	CHIKV-E1-AS1	10790–10768	TTYAYMGCTCTTACCGGGTTTGT	480
	CHIKV-E1-AS1B	10787–10768	AYCGCTCTTACCGGGTTTGT	200
2nd round (536 bp)	CHIKV-E1-S2	10228–10248	GGMGTCTACCCATTYATGTGG	400
	CHIKV-E1-S2B	10226–10248	CTGGMGTCTACCCATTTATGTGG	200
	CHIKV-E1-AS2	10763–10744	ATTTGRCAGCCRAAHGGTGC	600

E2 assay				
1st round (438 bp)	CHIKV-E2-S1	8973–8993	CCTTGCAGCACNTAYGYGCA	600
	CHIKV-E2-AS1	9410–9391	AGTGTTGGGTGRTCVGGRTA	600
2nd round (365 bp)	CHIKV-E2-S2	9046–9065	CCAGAYACYCCWGAYCRCAC	600
	CHIKV-E2-AS1	9410–9391	AGTGTTGGGTGRTCVGGRTA	600

a Assay sizes and primer positions are shown relative to a CHIKV reference genome (GenBank accession no. MG967666).

b H = A/C/T, M = A/C, N = A/C/G/T, R = A/G, W = A/T, V = A/C/G, and Y = C/T.

10.1128/mSphere.00295-19.1 FIG S1 Potential primer mismatches of reference and new assays. Mismatch analyses were conducted in Geneious 9.1.8. All sequences covering at least the full coding region were extracted from the complete alignment used for primer design and were applied to generate three new genotype-specific CHIKV alignments. Those genotype-specific alignments were used to identify mismatches. Genotype-specific potential mismatches with 100% consensus sequences are indicated in yellow, and those with 99% consensus sequences are indicated in red. General positions of potential mismatches are indicated by asterisks. Download FIG S1, TIF file, 2.7 MB. Copyright © 2019 Fischer et al. 2019 Fischer et al. This content is distributed under the terms of the Creative Commons Attribution 4.0 International license.

In contrast to reference assays, our new assays do not contain critical mismatches to circulating CHIKV variants under oligonucleotide binding sites (Fig. S1). Targeting highly conserved regions ensured broad usage of our assays across CHIKV genotypes but may increase the risk of amplifying other alphaviruses that are genetically related to CHIKV. Although of low relevance for molecular surveillance, we assessed the specificity of the new assays by testing nine heterologous alphaviruses representing all alphavirus serocomplexes. The new E1-based assay showed amplification of Mayaro (MAYV) and O'nyong-nyong virus (ONNV) in both rounds. In contrast, the new E2-based assay was specific for CHIKV and showed no amplification of other alphaviruses. These data were consistent with high similarity of CHIKV, MAYV, and ONNV under the E1-based assay oligonucleotide domains, whereas the oligonucleotide domains of the E2-based assay were less conserved among these alphaviruses (Fig. 2C).

### Conclusions.

Molecular epidemiological surveillance of circulating CHIKV strains is required for timely detection of adaptive mutations, which is crucial for risk assessments and efficient vector control (15). This need is underlined by the recent emergence of a new ECSA lineage that causes large outbreaks in Kenya, India, Pakistan, and Bangladesh. In contrast to the CHIKV ECSA IOL adapted to Aedes albopictus, the new ECSA lineage carries two new adaptive substitutions that improve transmission by Aedes aegypti (12). Those new adaptive mutations are located in the E1 and E2 domain hot spots covered by our new assays, suggesting the robustness of our assays for future adaptive mutations. The newly developed assays enhance molecular epidemiological surveillance of CHIKV by the combination of high analytical sensitivity, robustness across all CHIKV genotypes, and improved coverage of adaptive mutations. The high sensitivity and specificity of the E2-based assay enable alternative usage for CHIKV diagnostics in resource-limited settings.

### Methods.

**(i) Primer design.** Primers were designed manually relying on a data set composed of 1,763 GenBank entries for CHIKV aligned using MAFFT in Geneious 9.1.8 after removal of ambiguous bases (https://www.geneious.com).

**(ii) PCR protocols.** First-round PCR assays were conducted in 25-μl reaction mixtures using the Superscript III one-step RT-PCR system (Thermo Fisher Scientific, Darmstadt, Germany). Reaction mixtures were set up with 5 μl RNA, 12.5 μl 2× reaction buffer, 1 μg nonacetylated bovine serum albumin, and 1 μl enzyme. Amplification involved 57°C for 30 min, followed by 95°C for 3 min and 45 cycles of 95°C for 15 s, 57°C for 30 s, and 72°C for 48 s. Second-round PCR was performed using Platinum *Taq* polymerase (Thermo Fisher Scientific) with 2.5 mM MgCl and 0.2 mM deoxynucleoside triphosphates (dNTPs). Twenty-five-microliter reaction mixtures were set up using 2.5 μl 10× reaction buffer plus 0.1 μl enzyme and adding 1 μl first-round PCR product as the template. Primers for genotype-specific boosting of sensitivity in the E1 assays were applied at lower concentrations (Table 1).

**(iii) Tested CHIKV genotypes.** To determine the performance of PCR assays, RNA of the Asian (KP003813), ECSA (MG208125), and West African (AY726732) genotypes was tested. Before testing, viral RNA was quantified using the WHO CHIKV standard and diluted to specific concentrations. The WHO CHIKV standard is composed of a heat-inactivated CHIKV ECSA strain (R91064) isolated from a patient who got infected in 2006 in India. The strain was acquired from the Paul Ehrlich Institute and was used as specified by the manufacturer. One IU is equivalent to one genome copy, and the official concentration was determined by quantification by 24 expert laboratories (25).

**(iv) Specificity testing.** To assess the specificity of the new RT-PCR assays, they were tested with RNA extracted from high-titer virus stocks in the range of 10^6^ to 10^7^ PFU per ml each. For specificity testing we included Barmah Forest virus, Eastern equine encephalitis virus, MAYV, ONNV, Ross river virus, Semliki Forest virus, Sindbis virus, Venezuelan equine encephalitis virus, and Western equine encephalitis virus.

**(v) Protein structure modeling.** Adaptive amino acid exchanges were manually introduced into the translated sequence of AM258992 and modeled on a published crystal structure (PDB ID 3N40) using SWISS-MODEL (https://swissmodel.expasy.org/). Models were visualized with Chimera 1.13.1 (https://www.cgl.ucsf.edu/chimera/).
