# Supplementary figures and images for: Enhanced Molecular Surveillance of Chikungunya Virus
Source: mSphere. 2019 Jul 3;4(4):e00295-19. doi: 10.1128/mSphere.00295-19 (PMC6609226; doi:10.1128/mSphere.00295-19)

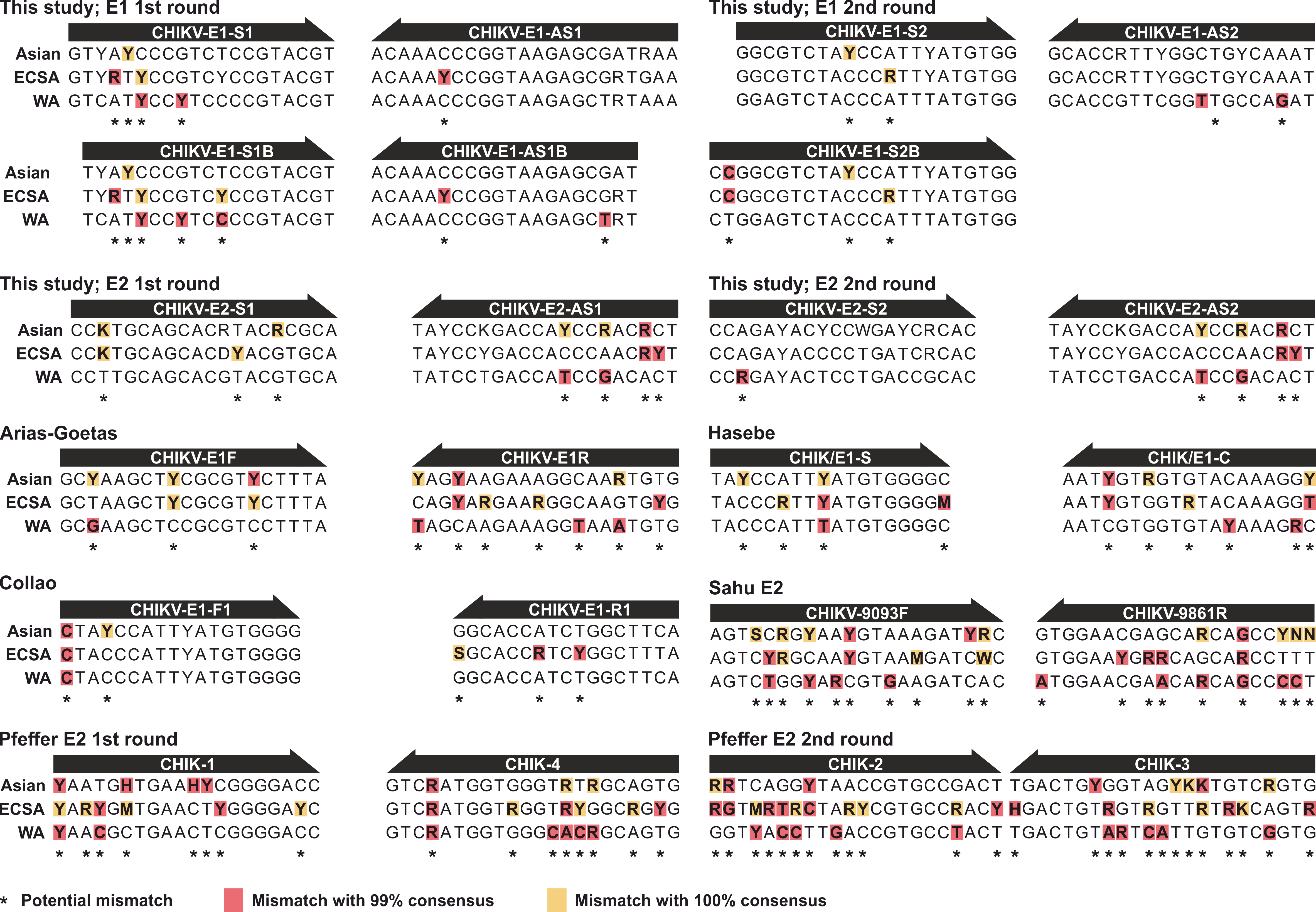

Supplement: FIG S1 [file mSphere.00295-19-sf001.tif]
